# Development of lentil peptides with potent antioxidant, antihypertensive, and antidiabetic activities along with umami taste

**DOI:** 10.1002/fsn3.3279

**Published:** 2023-02-27

**Authors:** Amir Rezvankhah, Mohammad Saeid Yarmand, Babak Ghanbarzadeh, Homaira Mirzaee

**Affiliations:** ^1^ Department of Food Science and Technology, Razi Food Chemistry Lab College of Agriculture and Natural Resources, University of Tehran Karaj Iran; ^2^ Department of Food Science and Technology, Faculty of Agriculture University of Tabriz Tabriz Iran; ^3^ Department of Food Science and Technology, Faculty of Agriculture Tarbiat Modares University Tehran Iran

**Keywords:** antioxidant, antihypertension, and antidiabetic activities, cross‐linking and sonication, lentil peptides, umami taste development

## Abstract

Lentil peptides have shown promising bioactive properties regarding the antioxidant activity and also inhibitory activity of angiotensin‐I‐converting enzyme (ACE). Sequential hydrolysis of proteins has shown a higher degree of hydrolysis with enhanced antioxidant and ACE‐inhibitory activities. The lentil protein concentrate (LPC) was sequentially hydrolyzed using Alcalase and Flavourzyme at 2% w/w. The hydrolysate (LPH) was cross‐linked (LPHC) or sonicated (LPHUS) and sequentially cross‐linked (LPHUSC). Amino acid profile, molecular weight (MW) distribution, DPPH and ABTS radical scavenging activities (RSA; 7 mg/mL), ACE (0.1–2 mg/mL), α‐glucosidase, and α‐amylase inhibitory activities (10–500 μg/mL), and umami taste were determined. The highest DPPH RSA was obtained for LPH (68.75%), followed by LPHUSC (67.60%), and LPHUS (67.49%) while the highest ABTS RSA values were obtained for LPHC (97.28%) and LPHUSC (97.20%). Cross‐linking and sonication led to the improvement of the ACE‐inhibitory activity so that LPHUSC and LPHC had IC_50_ values of 0.23 and 0.27 mg/mL, respectively. LPHC and LPHUSC also indicated higher α‐glucosidase inhibitory activity (IC50 of 1.2 and 1.23 mg/mL) compared to LPH (IC50 of 1.74 mg/mL) and LPHUS (IC50 of 1.75 mg/mL) while the IC50 value of acarbose indicated 0.51 mg/mL. Moreover, LPHC and LPHUSC exhibited higher α‐amylase inhibitory activities (IC50 of 1.35 and 1.16 mg/mL) than LPHUS (IC50 of 1.95 mg/mL), and LPH (IC50 of 2.51 mg/mL) while acarbose had an IC50 value of 0.43 mg/mL. Umami taste analysis revealed that LPH and LPHC due to MW of 1.7 and 2.3 kDa and also high umami amino acids could be well considered as representative of meaty and umami analog flavors while indicating stronger antioxidant, antihypertension, and antidiabetic attributes.

## Graphical abstract

Bioactive peptides were developed using enzymes. The produced peptides indicated potent antioxidant, antihypertensive, and antidiabetic activities. The produced peptides had high score umami tastes.

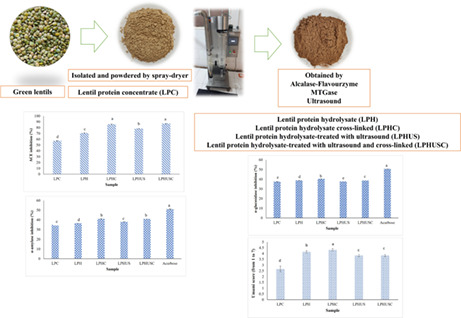

## 1 INTRODUCTION

Plant proteins have attracted great attention for their nutritional and pharmaceutical attributes (Emam‐Djomeh & Rezvankhah, 2020; Mirzaee et al., 2022; Rezvankhah et al., 2019, 2021a, 2021b; Rezvankhah, Yarmand, & Ghanbarzadeh, 2022). Many diseases have been widespread due to the consumption of artificial and synthetic additives in food formulations. Cardiovascular diseases, hypertension, cancers, type 2 diabetes, allergies, etc. are such health‐related concerns that even developed countries encounter (Elam et al., 2021; Fadimu, Gill, et al., 2022). Therefore, the intentions to use natural additives and health‐promoting compounds have been increased (Rezvankhah et al., 2018, 2021b; Rezvankhah, Emam‐Djomeh, et al., 2022). Plant proteins have natural peptide sequences that act as antioxidants, antihypertensive, and antidiabetic, and also participate in flavors such as umami and meaty (Fadimu, Farahnaky, et al., 2022; Fadimu, Gill, et al., 2022; Yan et al., 2021). However, the identification of these sequences from native proteins needs enough data and knowledge.

Enzymatic hydrolysis has been exploited to produce bioactive peptides with various health effects since 1970 (Dong et al., 2020; Lee & Hur, 2017). Different proteolytic enzymes have been used to produce bioactive peptides with multiple health and commercial performances (Sessa et al., 2014; Song et al., 2020; Wang et al., 2021). Alcalase and Flavourzyme are proteolytic enzymes with industrial applications (Rezvankhah et al., 2021a; Wei et al., 2018). Alcalase acts as an endopeptidase and cleaves the polypeptide chain from the inner section while Flavourzyme as an endo‐ and exopeptidase which cleaves the polypeptide chain from the end and inner sections (Gomes & Kurozawa, 2020; Xu et al., 2020). Alcalase‐mediated hydrolysis has been reported to have a higher degree of hydrolysis than Flavourzyme‐mediated hydrolysis (Ghelich et al., 2022; Xu et al., 2020). The peptides produced by Alcalase have bitter, umami, and meaty tastes while the peptides produced by Flavourzyme have sweet, umami, and meaty tastes (Fu et al., 2018; Rezvankhah et al., 2021a). Both enzymes are used in food flavor companies to develop umami and meaty tastes. Alongside these commercial applications, the peptides produced by these enzymes have shown strong antioxidant, antihypertension, and antidiabetic activities (Fadimu et al., 2021; Ozón et al., 2022).

Concerning the production of potent antioxidant, antihypertensive, and antidiabetic peptides, applying a dual‐enzyme system has been focused (Rezvankhah et al., 2021b). Indeed, the first enzyme cleaves the protein chains within the molecule and the second enzyme breaks peptide bonds from the endpiece of terminal amino acids of the peptides initially formed, thus producing lower molecular weight (LMW) peptides with higher biological activities (Ozón et al., 2022). The hydrolysates produced with dual‐enzyme system have shown stronger antioxidant, antihypertensive, and antidiabetic activities than hydrolysates produced by a single hydrolysis method (Lu et al., 2021; Rezvankhah et al., 2021b). Alcalase and Flavourzyme have been predominantly used in previous studies to produce potent bioactive peptides (Fadimu, Gill, et al., 2022; Rezvankhah, Yarmand, & Ghanbarzadeh, 2022).

Some studies have demonstrated that cross‐linking mediated by microbial transglutaminase (MTGase) and also ultrasound treatment of plant protein hydrolysates can increase the antioxidant, antihypertensive, and antidiabetic activities of former hydrolysates (Dabbour et al., 2020; Fadimu, Farahnaky, et al., 2022; Hayta et al., 2020; Jiang et al., 2018; Tian et al., 2020; Zhang, Huang, et al., 2021).

Lentil protein has been known as a strong antioxidant, antihypertensive, antidiabetic, and umami flavor source. It is a good plant protein substitute for animal protein sources especially when its protein is modified (Barbana et al., 2011; Barbana & Boye, 2011; Rezvankhah et al., 2021b). However, few studies have investigated the enzymatic hydrolysis, cross‐linking, and ultrasound effects on the lentil protein's bioactive and sensory properties (Boye et al., 2010; Rezvankhah et al., 2021a).

The objective of this study was the production of bioactive lentil protein hydrolysates by Alcalase and Flavourzyme and then modification of produced peptides by cross‐linking and sonication. The whole‐produced lentil protein hydrolysates were assessed for antioxidant, antihypertensive, and antidiabetic activities, and also umami taste development. No research studies were found regarding the generation of lentil peptides with various health and industrial uses.

## 2 MATERIALS AND METHODS

### 2.1 Materials

Lentil protein concentrate (LPC) with a protein content of 78% (w/w) was used. Alcalase (extracted from *Bacillus licheniformis*) and Flavourzyme (extracted from *Aspergillus oryzae*) were purchased from Novozymes Co. Microbial transglutaminase (MTGase) was prepared from Ajinomoto Co. Angiotensin‐I‐converting enzyme (ACE, extracted from rabbit lung) and the ACE synthetic substrate hippuryl‐l‐histidyl‐l‐leucine (HHL) were purchased from Sigma‐Aldrich Canada Ltd. Porcine pancreatic α‐amylase (Cat no. A3176) and rat intestinal α‐glucosidase (Cat no. I1630) were provided by Sigma‐Aldrich Co. All chemicals used in the present study were prepared by Sigma‐Aldrich Co. and had an analytical grade.

### 2.2 Hydrolysis of LPC

The heated LPC (3%, w/w, denatured at 90°C for 30 min) was hydrolyzed in a two‐stage process (Rezvankhah et al., 2021a). Alcalase and Flavourzyme were sequentially used at their specific optimum conditions. First, LPC's hydrolysis was initiated by Alcalase at 2% (w/w) enzyme to the substrate (E:S), the temperature of 60°C, and pH of 8.0. The first hydrolysis stage was accomplished for 120 min. Then, the Alcalase was deactivated by heating the protein/peptide solution at 90°C for 15 min. The protein/peptide solution was set at the optimum condition of Flavourzyme (temperature of 50°C and pH of 7.0) and the enzyme was added at 2% (w/w) E:S. The hydrolysis reaction continued for a further 60 min by Flavourzyme to reach the whole sequential hydrolysis time of 180 min. The enzyme was deactivated by heating at 90°C for 15 min. The peptide solution was centrifuged at 15,000 *g* for 15 min to separate the peptides from unhydrolyzed or insoluble proteins. The obtained supernatant was powdered using a mini spray dryer at inlet and outlet temperatures of 160 and 80°C, respectively, and air compressor pressure of 0.3–0.4 MPa. The obtained powder was termed lentil protein hydrolysate (LPH) and transferred to containers stored at 4°C. The degree of hydrolysis (DH) was determined using the method of Rezvankhah et al. (2021b) as below:

(1) $$\mathrm{DH}\;\left(\%\right)=B\times N_{b}\times \frac{1}{\alpha }\times \frac{1}{M_{p}}\times \frac{1}{h_{\mathrm{tot}}}$$

where *B* is the volume of consumed NaOH solution; *N*_*b*_ is the normality of basic solution; *M*_*p*_ is the mass of protein in LPC powder; *h*_tot_ is the total number of peptide bonds in the protein substrate (considered 7.78); *α* is the NH_2_ groups released during the hydrolysis and computed using the Equation below:

(2) $$\alpha =\frac{10^{\mathrm{pH}-\mathrm{pk}}}{1+10^{\mathrm{pH}-\mathrm{pk}}}$$

where pK is the average pK‐value of α‐NH_2_ groups liberated. pH is the optimum pH activity of enzymes used in hydrolysis. The pK is the temperature‐dependent parameter and is calculated by the following relation:

(3) $$\mathrm{pk}=7.8+\left(\frac{298-T}{298T}\right)\times 2400$$

where *T* (Kelvin, K) is the temperature of enzymatic hydrolysis.

### 2.3 Cross‐linking of LPH

The LPH prepared was treated with MTGase according to the method by Song et al. (2013) with slight modifications. The LPH (10%, w/w) solution was treated with MTGase for 5 h at E:S of 1.2% (w/w), pH of 8.0, and temperature of 45°C. After the cross‐linking process, the solution was heated at 95°C for 10 min for enzyme inactivation. The solution was centrifuged at 11,300 *g* for 10 min (25°C) and supernatant related to cross‐linked peptides was separated and powdered using a spray dryer. The obtained powder was called lentil protein hydrolysate cross‐linked (LPHC). The sample was stored at 4°C for further analysis.

### 2.4 Ultrasound treatment of LPH

Ultrasound treatment of LPH was performed according to the method of Tian et al. (2020) with slight modifications. LPH solution at 10% (w/w) was treated with an ultrasound probe system (TopSonics UPH‐400), 400 W output power and 20 kHz frequency, an amplitude range of 83–95 μm, and a flat tip probe diameter of 12 mm. The ultrasonic treatment was carried out at 150 W for 10 min at 2 s on and 2 s off conditions. The temperature of the process was controlled by an ice bath. The treated solution was centrifuged at 10,000 *g* for 20 min and the supernatant was collected and powdered using a spray dryer. The ultrasonic‐treated LPH was termed lentil protein hydrolysate ultrasound (LPHUS). Also, the produced LPHUS was cross‐linked with MTGase and termed lentil protein hydrolysate ultrasound cross‐link (LPHUSC).

### 2.5 Amino acid analysis

The amino acid profiles were determined using reversed‐phase high‐performance liquid chromatography (RP‐HPLC, Agilent 1100 HPLC; Agilent Ltd.), as reported by Rezvankhah et al. (2021a). First, the samples were hydrolyzed in glass tubes using 6 M HCl at 120°C for 12 h. Thereafter, the digests were filtered through a 0.22 μm pore size filter. The separation was performed using a Zorbax analytical column (C18, 4 × 250 mm, 5 μm particle size; Agilent) at the temperature of 40°C with an Ultraviolet detector spectra monitored at 338 nm. The elution of the column with the flow rate of 1 mL/min was conducted with mobile phases comprising 7.40 mmol/L of sodium acetate/triethylamine/tetrahydrofuran (400:0.10:2, v/v/v), set at pH 7.1 using acetic acid and 7.40 mmol/L of sodium acetate/methanol/acetonitrile (1.5:2.5:2.5, v/v/v), set at pH 7.1. A standard solution comprising 17 amino acids was used as an external standard.

### 2.6 MW profiles

MW profiles were determined by SDS–polyacrylamide gel electrophoresis (SDS‐PAGE) following the method reported by Rezvankhah et al. (2021b). First, a sample solution (5 mg/mL) of protein and produced hydrolysates was mixed with an equal amount of Laemmli sample buffer (960 μL of 66 mM Tris–HCl, pH 6.9, 27.3% w/v glycerol, 2.2% SDS, 0.01% bromophenol blue). Next, the prepared samples were combined with 2‐mercaptoethanol and heated for denaturation at 95°C for 5 min before the electrophoresis. The concentration of 12% Mini‐Protean™ precast gels (Bio‐Rad) was used to run the electrophoresis. Thereafter, 10 μL of cooled samples were loaded on the gels and then subjected to a constant voltage of 150 V. Additionally, a marker with MW standards (Bio‐Rad Broad Range Marker) was run alongside the samples. When the process finished, the gels were stained with 0.1% Coomassie Brilliant Blue R‐250 in a mixture of 10% acetic acid and 40% methanol for 2 h. The protein/peptide bands were visualized by discoloring the gels using a mixture of 40% methanol and 10% acetic acid solutions.

### 2.7 MW distribution

MW distribution of LP, LPH, LPC, LPHUS, and LPHUSC was studied according to the method of Rezvankhah et al. (2021b). Gel permeation chromatography system (GPC, Waters Breeze HPLC system, Waters Corporation) equipped with Waters Ultraviolet detector and Superdex Gel filtration column, phase Superdex Peptide HR (length × internal diameter 30 cm × 10 mm and 13–15 μm particle size) was used to determine MW distribution. The samples were dissolved in ultrapure water and then centrifuged at 12,000 *g* for 10 min. The supernatants were collected and filtered through a 0.22 μm membrane. The filtered solution was transferred into an analysis bottle and 50 μL of each sample was injected into the column. The isocratic elution process was considered for the column and 0.02 M phosphate buffer containing 0.25 M NaCl (pH 7.2) was used as the mobile phase at a flow rate of 0.5 mL/min. To determine the MW distribution of samples, the MW calibration curve was plotted using standards with specific retention times. The applied standards were cytochrome c, aprotinin, cyanocobalamin, glutathione disulfide, and glutathione reduced. The MW distribution of samples was obtained according to the comparison of the volume of the eluted peaks with the standard elution volumes.

### 2.8 Antioxidant activity

#### 2.8.1 DPPH radical scavenging activity

The antioxidant activity of hydrolysate samples was determined with DPPH radical scavenging activity using the method of Rezvankhah et al. (2021a). Samples at 7 mg/mL were prepared and 2 mL of each solution was mixed with 2 mL of DPPH ethanolic solution (0.2 mM). The obtained mixtures were placed in a dark place for 30 min to complete the scavenging of free radicals. The absorbance of solutions was measured using an Ultraviolet–vis spectrophotometer (SP‐UV 500DB spectrophotometer, Spectrum instruments). Ascorbic acid (0.01 mg/mL) was also used as the positive control. The DPPH radical scavenging activity was ascertained by Equation (4):

(4) $$\text{Radical scavenging activity}\;\left(\mathrm{RSA},\%\right)=\frac{A_{C}-A_{S}}{A_{C}-A_{B}}\times 100$$

where *A*_*C*_, *A*_*S*_, and *A*_*B*_ exhibits the absorbance values of control, sample, and blank, respectively.

#### 2.8.2 ABTS radical scavenging activity

The ABTS°^+^ scavenging activity of the protein and produced hydrolysates was measured according to the protocol reported by Mirzaee et al. (2022). The ABTS solution (940 μL) was combined with 60 μL of samples (7 mg/mL) and vigorously shaken, and then incubated at 25°C for 10 min in the dark. The absorbance was read at 734 nm using a spectrophotometer and ascorbic acid (0.01 mg/mL) was used as a positive control. ABTS RSA was calculated using Equation (4).

### 2.9 Antihypertensive activity

The ACE‐inhibitory activity of samples was determined based on the method of Ambigaipalan et al. (2015) with slight modifications. The HEPES‐HCl buffer (50 mM) containing 300 mM NaCl (adjusted to pH 8.3) was used for dissolving the samples and enzyme. The samples at 0.1–2 mg/mL were prepared and 10 μL of them was mixed with 20 μL of ACE solution (0.25 unit/mL). The obtained mixture was preincubated at 37°C for 5 min. The reaction was initiated by dissolving 50 μL of HHL (6 mg/mL) in distilled water and then added to the mixture followed by incubation at 37°C for 15 min. During the reaction, hippuric acid was formed and extracted with 1 mL of ethyl acetate. The mixture was centrifuged at 1200 *g* for 5 min and the separated supernatant was collected and transferred to boiling water to remove ethyl acetate. The retained hippuric acid was dissolved in 1 mL of distilled water and the absorbance was read at 228 nm. The control was prepared using 50 mM HEPES‐HCl buffer containing 300 mM NaCl (pH 8.3) instead of the sample. The sample blank and control blank were run in the same manner, except that ACE solution was incorporated into the reaction before the addition of 1 M HCl. The ACE‐inhibitory activity (%) was computed using Equation (5):

(5) $$\mathrm{ACE}-\text{inhibitory activity}\;\left(\%\right)=\frac{A_{C}-A_{S}}{A_{C}-A_{B}}\times 100$$

where *A*_*C*_, *A*_*S*_, and *A*_*B*_ indicate the absorbance values of control, sample, and blank, respectively. The serial concentration of 0.1–2 mg/mL was prepared to calculate the IC_50_ values.

### 2.10 Antidiabetic activity

#### 2.10.1 α‐Glucosidase inhibitory activity

The α‐glucosidase (rat intestinal) inhibitory activity of samples was determined according to the method described by Rezvankhah, Yarmand, and Ghanbarzadeh (2022) with slight modifications. The enzyme with an activity of 90 mU/mL was used. One hundred microliters of each sample solution (10–500 μg/mL; pH 6.9, in 6 mM NaCl) was mixed with 200 μL of α‐glucosidase and incubated at 37°C for 10 min. After preincubation, 100 μL of p‐nitrophenyl‐α‐d‐glucopyranoside (PNPG) solution (5 mM) was mixed with the prepared mixture and incubated at 37°C for 10 min. The absorbance of solution was determined every 2 min at 405 nm. The phosphate buffer (pH 6.8) was used as a control instead of sample solution. Also, acarbose was used as the positive control. The α‐glucosidase inhibitory activity was calculated by Equation (6):

(6) $$\alpha -\text{glucosidase inhibitory activity}\;\left(\%\right)=\frac{A_{C}-A_{S}}{A_{C}}\times 100$$

where *A*_*C*_ and *A*_*S*_ represent the absorbance of control and sample, respectively. IC_50_ values were determined based on the serial concentration (10–500 μg/mL).

#### 2.10.2 α‐Amylase inhibitory activity

The α‐amylase inhibitory activity was determined using the method of Rezvankhah, Yarmand, and Ghanbarzadeh (2022) with slight modifications. One hundred microliters of sample solution (10–500 μg/mL; pH 6.9, in 6 mM NaCl) was mixed with 100 μL of α‐amylase solution (0.5 U/mL) and incubated at 37°C for 5 min. After preincubation, 100 μL of starch solution (0.5%, w/v) was added to the prepared mixture followed by incubation at 37°C for 20 min. Then, the reaction mixture was heated at 100°C for 10 min and then cooled down to the ambient temperature followed by centrifuging at 15,000 *g* for 2 min to separate the undigested starch. Twenty microliters of supernatant were mixed with 1 mL of p‐hydroxybenzoic acid hydrazide (PAHBAH) and heated to 70°C for 10 min. The solution was cooled down to room temperature and the absorbance was read at 410 nm. Also, acarbose was used as the positive control. The α‐amylase inhibitory activity was determined using Equation (7):

(7) $$\alpha -\text{amylase inhibitory activity}\;\left(\%\right)=1-\frac{\left(A_{S}-A_{B}\right)}{A_{C}}\times 100$$

where *A*_*S*_, *A*_*B*_, and *A*_*C*_ show the absorbance values of sample, blank (phosphate buffer, enzyme, and sample), and control (starch, buffer, and enzyme), respectively. IC_50_ values were calculated by preparation of serial concentrations.

### 2.11 Umami sensory analysis

The umami taste evaluation was conducted according to the method reported by Rezvankhah et al. (2021a) with slight modifications. Six flavor specialists were hired for taste evaluation and asked to score the samples from 1 to 7 based on the 7‐point hedonic method. The samples including LPH, LPHC, LPHUS, and LPHUSC were dissolved in an umami soup at 1% w/v. The umami soup consisted of 0.5% w/v salt and 1% w/v monosodium glutamate. The semi‐taught panelists were asked to give scores to the test solutions. The reference sample was a mixture of LPC and umami soup.

### 2.12 Statistical analysis

All experiments were performed at three replications. The comparison of data mean difference was carried out by ANOVA and Duncan's test using SPSS software.

## 3 RESULTS AND DISCUSSION

### 3.1 DH of hydrolysate

The LPC was hydrolyzed using a two‐step method. First, LPC was hydrolyzed by Alcalase to reach a DH of 17.5%. Alcalase is an endopeptidase enzyme that extensively breaks down the peptide bonds from an interior section of proteins and releases peptides with low MW (Tacias‐Pascacio et al., 2020). Based on the previous reports, the produced peptides by Alcalase had medium and small MW sizes (Rezvankhah et al., 2021b; Tacias‐Pascacio et al., 2020). Also, Alcalase‐produced hydrolysates have shown a bitter taste which is mostly related to the release of peptides with hydrophobic segments and amino acids (de Carvalho et al., 2019; Tacias‐Pascacio et al., 2020). When the Alcalase was individually applied, the predominant peptides produced for LPC revealed MW of 10 kDa (Rezvankhah et al., 2021a, 2021b). These peptides have shown high potential for antioxidant and antihypertensive activities (Fadimu, Gill, et al., 2022). Following the study, the produced hydrolysate (LPH) was further hydrolyzed by Flavourzyme which has endo‐ and exopeptidase activities (Fadimu, Gill, et al., 2022; Rezvankhah et al., 2021a). It has been reported that the peptides produced by a single Flavourzyme have higher MW, indicating the lower DH of hydrolysates, and also lower bitterness compared to Alcalase‐produced hydrolysates (Rezvankhah et al., 2021a, 2021b). In the present study, DH was obtained at about 17.5% by Alcalase through the first step while reaching 36% by Flavourzyme after the second step (sequential hydrolysis). It was observed that the sequential hydrolysis of proteins can remarkably raise the DH, likely decrease the bitterness of generated peptides, and plausibly increase the antihypertensive, and antidiabetic activities (Aryee & Boye, 2016; Dabbour et al., 2020; Ozón et al., 2022; Rivero‐Pino et al., 2021). It was postulated that if the Flavourzyme had been used as the first enzyme, the DH did not reach such high value. This could be associated with that the Flavourzyme hydrolyzes the proteins from the exterior section of the protein and produces larger peptides and also free amino acids while Alcalase provides more substrates (high available peptide bonds) for Flavourzyme and thus, DH remarkably raises (Fadimu, Gill, et al., 2022; Hu et al., 2020; Rezvankhah, Yarmand, & Ghanbarzadeh, 2022; Xu et al., 2020). On the other side, Flavourzyme also has been well known to produce peptides with meaty and umami tastes (Fu et al., 2018; Wei et al., 2018).

### 3.2 Amino acid profile

The results of amino acid profiles of LPC, LPH, LPHC, LPHUS, and LPHUSC are presented in Table 1. Glutamic acid, aspartic acid, leucine, lysine, and arginine were abundantly found in the LPC and also the produced hydrolysates. The hydrolysis, cross‐linking, sonication, and combined sonication and cross‐linking led to some alterations in the amino acid composition possibly due to the separation of some nontreated peptides/polypeptides during the centrifugation process (Fadimu, Gill, et al., 2022; Mirzaee et al., 2022; Rezvankhah, Yarmand, & Ghanbarzadeh, 2022; Zhang, Cheng, et al., 2021). These changes were evidenced by the following amino acid residue yields: 96.29%, 97.19%, 98.29%, 96.17%, and 92.21% for LPC, LPH, LPHC, LPHUS, and LPHUSC, respectively. The order of hydrophobic amino acid content was LPHUS (39.79%), LPC (38.86), LPHC (38.79%), LPH (38.26), and LPHUSC (37.27%). The high hydrophobic amino acids of LPC could be attributed to its inherent hydrophobic segments, which have been buried within the protein spatial structure. After the hydrolysis, the buried hydrophobic parts are exposed and thus, the biological activities are altered (Rezvankhah et al., 2021b). Hydrolysis, cross‐linking, sonication, and combined sonication and cross‐linking also altered the charged amino acid contents (Table 1). These alterations could not only be attributed to the specificity of enzymes (Alcalase, Flavourzyme, and MTGase) that acted on the LPC, but the sonication could also affect the amino acid profiles by disrupting the aggregate of peptides (Fadimu, Gill, et al., 2022; Jia et al., 2010; Jin et al., 2015). Regarding the negatively charged amino acids, the order was LPH (30.08%), LPHC (29.44%), LPC (27.78%), LPHUS (27.06%), and LPHUSC (26.36%). The charged amino acids can increase the interaction of peptides with water‐soluble radicals (ABTS radicals), thus increasing the antioxidant power (Hu et al., 2020; Mirzaee et al., 2022; Rezvankhah et al., 2021a). In terms of amino acids with sulfur groups, the order was LPHC (3.35%), LPHUSC (3.34%), LPC (3.24%), LPHUS (3.11%), and LPH (2.77%). Sulfur‐containing amino acids can contribute to the meaty‐analog flavor and umami taste (Rezvankhah et al., 2021a; Wei et al., 2018). The umami taste has been introduced as a savory property and so palatable taste that originated from aspartic and glutamic acids (Großmann et al., 2021; Sonklin et al., 2018). The order of umami amino acids was LPHC (42.16%), LPH (41.81%), LPHUS (40.79%), LPHUSC (38.98%), and LPC (38.74%). Hence, LPHC and LPH could be introduced as natural flavor enhancers.

**TABLE 1:** RP‐HPLC amino acid profiles of LP, LPH, LPHC, LPHUS, and LPHUSC.

Amino acid composition (%w/w)	LP	LPH	LPHC	LPHUS	LPHUSC
Aspartic acid (Asp)	12.02	13.01	11.92	11.61	11.33
Serine (Ser)	5.35	4.59	5.49	5.23	4.98
Glutamic acid (Glu)	15.76	17.07	17.52	15.45	15.03
Glycine (Gly)	4.43	4.26	3.94	4.42	4.13
Histidine (His)	2.02	1.81	2.17	2.35	2.19
Arginine (Arg)	6.59	7.67	7.43	6.65	6.72
Threonine (Thr)	3.88	4.41	3.76	3.19	2.82
Proline (Pro)	4.73	5.40	5.81	6.28	5.54
Alanine (Ala)	4.21	4.52	4.74	5.10	4.89
Cystine (Cys)	1.51	1.30	1.44	1.31	1.61
Tyrosine (Tyr)	3.26	2.64	2.91	3.25	3.69
Valine (Val)	4.88	4.38	4.58	4.81	4.27
Methionine (Met)	1.73	1.47	1.91	1.80	1.73
Lysine (Lys)	8.56	7.73	8.32	8.65	8.19
Isoleucine (Ile)	3.86	3.45	3.60	3.11	2.87
Leucine (Leu)	8.98	9.35	9.17	9.76	9.70
Phenylalanine (Phe)	4.53	4.13	3.60	3.20	2.53
Amino acid residue yield (%)	96.29	97.19	98.29	96.17	92.21
Total essential amino acids	38.44	36.73	37.11	36.87	34.3
Total nonessential amino acids	57.86	60.46	61.2	59.3	57.92
Hydrophobic amino acids	38.86	38.26	38.79	39.79	37.27
Hydrophilic amino acids	14	12.94	13.6	12.98	13.1
Positively charged amino acids	17.17	17.21	17.92	17.65	17.1
Negatively charged amino acids	27.78	30.08	29.44	27.06	26.36
Sulfur‐containing amino acids	3.24	2.77	3.35	3.11	3.34
Umami amino acids	38.74	41.81	42.16	40.79	38.98

*Note*: Essential amino acids include His, Ile, Leu, Lys, Met, Phe, Thr, and Val. Nonessential amino acids include Asp, Ser, Glu, Gly, Arg, Pro, Ala, Cys, and Tyr. Hydrophobic amino acids include Ala, Val, Ile, Leu, Phe, Pro, Met, Cys, and Gly. Hydrophilic amino acids include Ser, Thr, Cys, and Tyr. Positively charged amino acids include Arg, His, and Lys. Negatively charged amino acids include Asp and Glu. Sulfur‐containing amino acids include Met and Cys. Umami amino acids include Asp, Glu, His, Pro, and Ala.

### 3.3 MW profile

**FIGURE 1:**
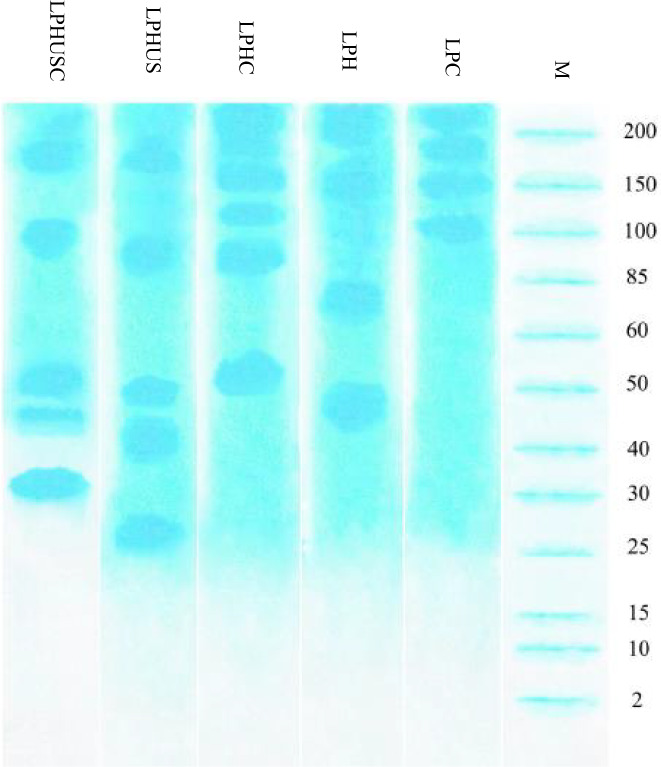
SDS‐PAGE patterns of LPC (control), LPH, LPHC, LPHUS, and LPHUSC. LPC was native lentil protein; LPH was Alcalase–Flavourzyme hydrolysates; LPHC was MTGase cross‐linked LPH; LPHUS was treated LPH with ultrasound; LPHUSC was LPH treated with ultrasound and MTGase.

MW profiles of LPC, LPH, LPHC, LPHUS, and LPHUSC are presented in Figure 1. For LPC, four intensive bands were detected at 100, 150, 170, and 200 kDa. The sequential hydrolysis led to releasing peptides with MW of 45 and 70–75 kDa (LPH) which was also reported by Boye et al. (2010) and Rezvankhah et al. (2021b). MTGase‐mediated cross‐linking of LPH had enlarging effects so that the mentioned bands were shifted to higher MW (50–55 and 88–90 kDa; LPHC). In contrast, sonication had disruptive effects, and the bands (70–75 and 45 kDa) were segregated into peptides with MWs of 50, 40–45, and 25–27 kDa (LPHUS). Also, sonication disrupted the peptides with high MW >100 kDa and formed a band at 88–90 kDa. The combination of sonication and cross‐linking indicated both segregated (due to ultrasonic treatment) and enlarged peptides (due to cross‐linking treatment) with MW of 50–52, 45–47, and 30–33 kDa (LPHUSC). Similar results were found for sonication and cross‐linking of plant protein hydrolysates (Fadimu et al., 2021; Fadimu, Farahnaky, et al., 2022; Yu et al., 2019; Zhang, Huang, et al., 2021).

### 3.4 MW distribution

**FIGURE 2:**
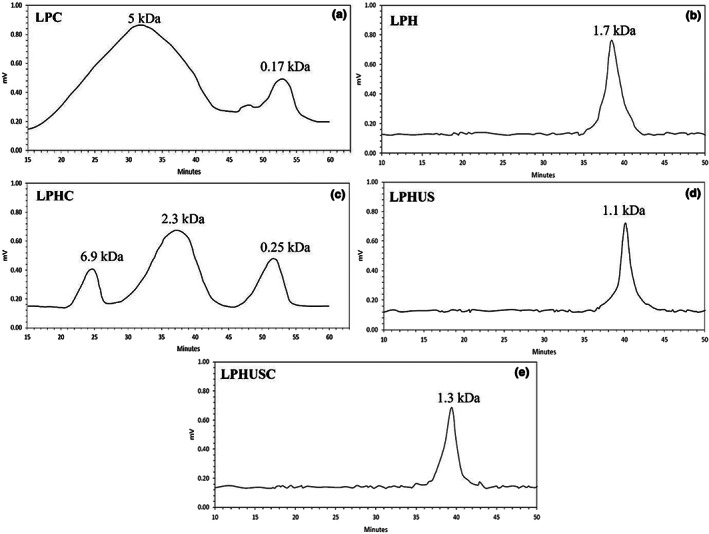
GPC analysis of MW distribution of LPC (control) (a), LPH (b), LPHC (c), LPHUS (d), and LPHUSC (e). LPC was native lentil protein; LPH was Alcalase–Flavourzyme hydrolysates; LPHC was MTGase cross‐linked LPH; LPHUS was treated LPH with ultrasound; LPHUSC was LPH treated with ultrasound and MTGase.

GPC analysis was used to estimate the MW distribution of produced peptides (Figure 2). The difference between the data obtained by electrophoresis and GPC is that the SDS‐PAGE has been reported to determine the MW of peptides/proteins higher than 10 kDa while GPC is an appropriate technique which is usually used to determine the MW lower than 10 kDa (Fadimu et al., 2021; Fadimu, Farahnaky, et al., 2022; Fadimu, Gill, et al., 2022). Accordingly, LPC indicated a broad peak that was assigned to peptides with MW of 5 kDa. It was in agreement with that obtained by Rezvankhah, Yarmand, and Ghanbarzadeh (2022). By hydrolysis, a sharp peak was obtained for LPH with MW of 1.7 kDa. This confirmed the hydrolysis and production of peptides with significant alteration in molecular size (Avramenko et al., 2013; Boye et al., 2010). When MTGase‐mediated cross‐linking was exerted on LPH, clear enlarging of peptides was observed (Figure 2). For LPHC, a relatively short peak was detected at MW of 6.9 kDa, a broad peak was detected at 2.3 kDa, and also a peak at MW of 0.25 kDa. Indeed, cross‐linking enlarged the produced peptides during the hydrolysis and increased the peptides with MW of 2.3 kDa, which have been introduced as the most contributing peptides in umami taste (Fu et al., 2018; Großmann et al., 2021; Rezvankhah et al., 2021b; Sonklin et al., 2018; Zhao et al., 2019). In contrast, sonication of LPH led to the disruption of aggregated peptides formed during the hydrolysis, and peptides with low MW (1.1 kDa) were liberated for LPHUS (Jin et al., 2015; Jin, Ma, et al., 2016). The combination of ultrasonic and cross‐linking treatments (LPHUSC) led to the formation of peptides with MW of 1.3 kDa which was lower than LPH and LPHC while higher than LPHUS (Figure 2).

### 3.5 Antioxidant activity

The produced LPH was subjected to MTGase‐mediated cross‐linking and ultrasound treatment aiming that strong antioxidant hydrolysates might be produced. Hence, the DPPH RSA of modified LPH was determined. DPPH radicals have been reported to be oil soluble, efficient interacting with hydrophobic antioxidants (Hu et al., 2020; Rezvankhah et al., 2021b). LPH due to high concentration of exposed hydrophobic amino acid residues such as alanine, valine, isoleucine, leucine, phenylalanine, proline, methionine, cysteine, and glycine can potentially interact with hydrophobic DPPH radicals (Table 1; Rezvankhah et al., 2021a, 2021b; Sonklin et al., 2018; Tian et al., 2022).

**FIGURE 3:**
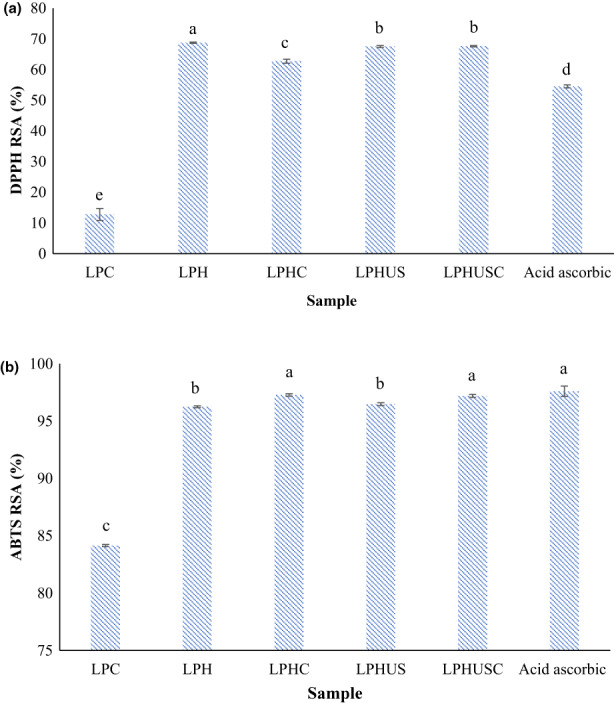
DPPH RSA (a) and ABTS RSA (b) of LPC, sequentially hydrolyzed LPC (LPH), cross‐linked LPH (LPHC), sonicated LPH (LPHUS), and sonicated cross‐linked LPH (LPHUSC). Acid ascorbic was used as positive control. The concentration of protein and hydrolysates were 7 mg/mL while it was 0.01 mg/mL for ascorbic acid.

According to Figure 3a, LPC as control indicated 12.77% DPPH RSA (at 7 mg/mL). The antioxidant activity of LPC could be associated with antioxidant amino acid residues which can donate hydrogen atoms to free radicals (DPPH radicals; Garcia‐Mora et al., 2014; Rezvankhah et al., 2021a). The antioxidant amino acids are aspartic acid, glutamic acid, proline, arginine, methionine, leucine, alanine, tyrosine, and valine (Table 1; Avramenko et al., 2013; Hu et al., 2022; Qiao et al., 2020). In addition, phenolic compounds present in plants are covalently bound with proteins, thus contributing to antioxidant activity (Amini Sarteshnizi et al., 2021; Hernández‐Jabalera et al., 2015).

When LPC was hydrolyzed sequentially using Alcalase and Flavourzyme, DPPH RSA was significantly increased to 68.75% for LPH (at 7 mg/mL; Figure 3a; *p* < .05). This increase could be related to the exposure of hydrophobic peptides buried in the interior structure of the protein (Avramenko et al., 2013; Rezvankhah, Yarmand, & Ghanbarzadeh, 2022). Denaturation and subsequent enzymatic hydrolysis unfolded the protein by altering its third, second, and first structures (Habinshuti et al., 2019; Liu et al., 2012; Wang et al., 2021). These conformation alterations led to exposing the hydrophobic peptides to the surface, increasing the interactions with DPPH radicals (Wang et al., 2021). Besides, small‐ and medium‐sized peptides have high potential in donating hydrogen atoms to interactive oxidizing radicals (Jin, Liu, et al., 2016; Wang et al., 2021). Presumably, the exposed hydrophobic segments with hydrophobic amino acid residues could likely increase the interaction of peptides with hydrophobic DPPH radicals (Jin et al., 2015). Hence, LPH with DH of 36% and lower MW, and also exposed structure with high hydrophobic amino acids residues strongly contributed to the antioxidant activity (Jin et al., 2015; Rezvankhah, Yarmand, & Ghanbarzadeh, 2022).

Regarding the cross‐linking mediated by MTGase (LPHC), a reduction of DPPH RSA (62.71% at 7 mg/mL) was observed possibly due to a reduction of surface hydrophobicity (Figure 3a; *p* < .05; Quintero‐Soto et al., 2021; Rezvankhah, Yarmand, & Ghanbarzadeh, 2022; Song et al., 2013). Indeed, cross‐linking reburied the hydrophobic peptides formerly exposed to the surface of hydrolysate molecules, thus decreasing the reactivity with DPPH radicals (He et al., 2021; Rezvankhah, Yarmand, & Ghanbarzadeh, 2022). A similar explanation has been reported in previous studies (Jin et al., 2015; Jin, Liu, et al., 2016; Liu et al., 2015). Cross‐linking reaggregated the hydrolysate molecules, thus the availability of exposed hydrophobic segments and also the interaction with DPPH radicals were decreased (Quintero‐Soto et al., 2021; Song et al., 2013).

Ultrasound treatment (LPHUS) and combined ultrasound and cross‐linking (LPHUSC) slightly decreased the DPPH RSA compared to LPH, showing 67.49% and 67.60% DPPH RSA (at 7 mg/mL), respectively (Figure 3a; *p* < .05). Sonication has been reported to disrupt the aggregated hydrolysate molecules formed in the hydrolysis process (Tian et al., 2020; Zhang, Huang, et al., 2021). These slight reductions for LPHUS and LPHUSC could be also associated with the liberation of peptides with charged amino acids (negatively and positively) which have low interaction with hydrophobic DPPH radicals (Jin, Ma, et al., 2016; Tian et al., 2020).

Regarding the ABTS RSA (Figure 3b), treatments significantly altered the antioxidant activity (*p* < .05). LPC indicated 84.14% RSA while LPH, LPHC, LPHUS, LPHUSC, and ascorbic acid indicated 96.25%, 97.28%, 96.48%, 97.20%, and 97.6% RSA, respectively. The increase in ABTS RSA could be related to the liberation of peptides with altered negatively and positively charged amino acids based on the amino acids analysis in Table 1. It has been reported that ABTS radicals are water soluble and efficiently interact with hydrophilic peptides with negatively or positively charged amino acid residues (Hu et al., 2020; Mirzaee et al., 2022).

### 3.6 ACE‐inhibitory activity

**FIGURE 4:**
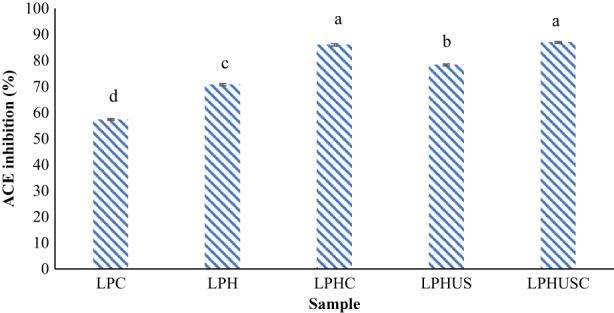
ACE inhibition of LPC, hydrolyzed LPC (LPH), cross‐linked LPH (LPHC), sonicated LPH (LPHUS), and sonicated cross‐linked LPH (LPHUSC). The concentration of protein and hydrolysates was 2 mg/mL.

ACE is an enzyme that contributes to the increase of blood pressure, leading to cardiovascular diseases (Lu et al., 2021; Xie et al., 2019). Prevention of the activity of this enzyme is so crucial regarding hypertension control (Mirzaee et al., 2022). The ACE‐inhibitory activity of lentil proteins and their hydrolysates have been reported by several authors (Akillioǧlu & Karakaya, 2009; Boye et al., 2010). According to Figure 4, LPC exhibited an ACE inhibitory activity of 57.35% for the concentration of 2 mg/mL which evidenced the presence of peptides with ACE inhibitory activity in LPC. Hydrolysis of LPC led to a significant increase in ACE‐inhibitory activity and increased to 70.73% for LPH (at 2 mg/mL; *p* < .05). The liberation of small‐ and medium‐sized peptides supported the strong ACE‐inhibitory activity such as the legumin, albumin, and vicilin fractions that have been reported to be major responsible for ACE inhibition (Garcia‐Mora et al., 2014, 2015; He et al., 2013; Rezvankhah et al., 2021a, 2021b).

Using MTGase for cross‐linking of LPH resulted in a significant increase of ACE‐inhibitory activity, reaching 86% for LPHC (at 2 mg/mL; Figure 4; *p* < .05). Reaggregation of some peptides gave rise to higher ACE inhibition where peptides with MW in the range of 3.5–7 kDa have shown high potential to inhibit the ACE activity (Jakubczyk et al., 2017; Quintero‐Soto et al., 2021; Zhang, Cheng, et al., 2021). Similarly, researchers also have suggested that the association of peptides by cross‐linking led to stronger ACE‐inhibitory activity, which might be attributed to the generation of peptides with the presence of hydrophobic amino acid residues (Quintero‐Soto et al., 2021; Rezvankhah, Yarmand, & Ghanbarzadeh, 2022; Xie et al., 2019; Yu et al., 2019). Lentil protein and its hydrolysates have shown higher hydrophobic amino acids which confer high biological activities (Barbana & Boye, 2011; Xie et al., 2019).

Sonication of LPH resulted in an increment of ACE‐inhibitory activity, reaching 78.32% (2 mg/mL) for LPHUS, higher than LPH (*p* < .05). Sonication might produce peptides with a specific MW range. These peptides due to disruption and microstreaming effects of sonication were disintegrated and the robust biological segments were exposed, indicating higher antihypertension activity (Dabbour et al., 2020; Fadimu, Farahnaky, et al., 2022).

Combined effects of sonication and cross‐linking were also investigated for ACE‐inhibitory activity alterations. The combined treatments of sonication and cross‐linking led to the obtaining of the highest ACE inhibition (86.96% at 2 mg/mL) with no remarkable difference with LPHC (86%; *p* > .05).

IC_50_ values of samples were determined as the concentration of peptide/protein which could inhibit 50% of ACE activity. The respective values were 0.91 ± 0.05, 0.48 ± 0.02, 0.27 ± 0.04, 0.33 ± 0.03, and 0.23 ± 0.04 mg/mL for LPC, LPH, LPHC, LPHUS, and LPHUSC, respectively (Table 2). Based on the applied enzymes and also DH values, ACE inhibitory can also be altered. Lower MW peptides with high hydrophobic amino acids have shown stronger ACE‐inhibitory activity (Boye et al., 2010; Garcia‐Mora et al., 2014). IC_50_ values of 0.15–0.44 mg/mL have been reported for LPH (Akillioǧlu & Karakaya, 2009; Boye et al., 2010; Garcia‐Mora et al., 2014; Rezvankhah et al., 2021b).

**TABLE 2:** IC_50_ values for ACE, α‐glucosidase, and α‐amylase inhibitory activities.

Sample	IC_50_ of inhibitory activity		
	ACE (mg/mL)	α‐Glucosidase (mg/mL)	α‐Amylase (mg/mL)
LPC	0.91 ± 0.05^a^ ^†^	2.58 ± 0.05^a^	4.2 ± 0.12^a^
LPH	0.48 ± 0.02^b^	1.74 ± 0.09^b^	2.5 ± 0.08^b^
LPHC	0.27 ± 0.04^cd^	1.20 ± 0.01^c^	1.35 ± 0.07^d^
LPHUS	0.33 ± 0.03^c^	1.75 ± 0.06^b^	1.95 ± 0.11^c^
LPHUSC	0.23 ± 0.04^d^	1.23 ± 0.03^c^	1.16 ± 0.05 ^e^
Acarbose	–	0.51 ± 0.07^d^	0.43 ± 0.02^f^

^†^ The different small superscripts indicate a statistically significant difference between the columns (*p* < .05).

ACE like other enzymes has binding sites that could interact with potent peptides (Quintero‐Soto et al., 2021). The higher interactions of peptides and ACE are formed, so stronger inhibitory activity can be delivered (Lu et al., 2021). Also, the lower binding energy is needed for interactions, indicating a high affinity between the peptides and ACE (Lu et al., 2021). The hydrophobic amino acids located on the enzyme's active site allow the interaction of uncharged amino acids (Karimi et al., 2020; Lu et al., 2021). Regarding the dipeptidyl peptidase‐4 (DPP4) as an enzyme with a similar role in hypertension, it has been reported two binding sites S1 and S2 (Quintero‐Soto et al., 2021). S1 consists of hydrophobic amino acids (TYR^547^, TYR^631^, VAL^656^, TRP^659^, TYR^662^, TYR^666^, and VAL^711^) that interact with uncharged amino acids including SER^630^, ASP^708^/ASN^710^, and HIS^740^ as catalytic triad (Quintero‐Soto et al., 2021). S2 involves key interactions with GLU^205^, GLU^206^, and ARG^125^, and is made up of a long hydrophobic chain and an aromatic chain that increases the enzyme‐substrate affinity (Quintero‐Soto et al., 2021). Hence, LPHC and LPHUSC possibly due to their hydrophobic segments and amino acids had the most efficient inhibition impacts on the ACE activity (Table 1).

### 3.7 Antidiabetic activity

Diabetes type 2 is also called non‐insulin‐dependent diabetes mellitus that has been a widespread disease (Chandrasekaran & Gonzalez de Mejia, 2022; Fadimu, Gill, et al., 2022; Jakubczyk et al., 2017). When the patient uses starches composed of amylose and amylopectin, maltose, lactose, sucrose, etc., they are degraded into small units, meaning glucose molecules, and then absorbed into the blood by the adjacent cells of the gastrointestinal tract, increasing the blood glucose level (Mirzaee et al., 2022; Rezvankhah, Yarmand, & Ghanbarzadeh, 2022). The increase in blood glucose levels causes other disorders such as coronary heart disease, high blood pressure, etc. (Fadimu, Gill, et al., 2022; Rahimi et al., 2022). Thus, the conversion of starches and disaccharides to smaller units should be inhibited using drugs such as acarbose and voglibose (Ramírez Fuentes et al., 2021). These drugs by inhibition of α‐glucosidase and α‐amylase exert antidiabetic activities (Arise et al., 2019; Fadimu, Farahnaky, et al., 2022). It is worth to mentioning that α‐glucosidase can just break down the α‐1,4 bonds in disaccharide molecules and release glucose while α‐amylase cleaves the α‐1,4 and α‐1,6 bonds present in starch molecules and releases more glucose molecules (Liu et al., 2021; Mirzaee et al., 2022; Rezvankhah, Emam‐Djomeh, et al., 2022; Rezvankhah, Yarmand, & Ghanbarzadeh, 2022). The commercial inhibitor (antidiabetic drugs) efficiently inhibit enzyme activity and the production of glucose molecules is limited. However, chemical drugs have shown side clinical problems such as diarrhea and flatulence (Chandrasekaran & Gonzalez de Mejia, 2022; Rezvankhah, Yarmand, & Ghanbarzadeh, 2022). In contrast, bioactive peptides derived from plant proteins have demonstrated good antidiabetic activities, indicating inhibition impacts on the α‐glucosidase and α‐amylase by substituting at the active sites, reducing the glucose production (Kamal et al., 2021; Ramírez Fuentes et al., 2021).

#### 3.7.1 α‐Glucosidase inhibitory activity

**FIGURE 5:**
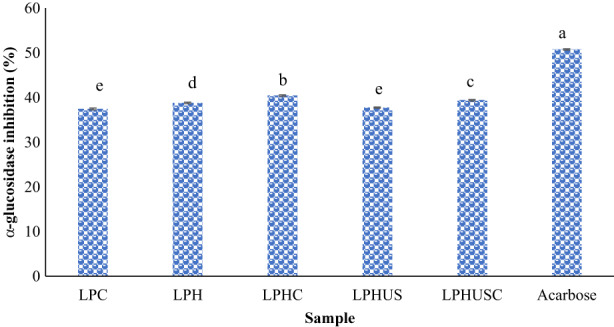
α‐glucosidase inhibition of LPC, hydrolyzed LPC (LPH), sonicated LPH (LPHUS), and sonicated cross‐linked LPH (LPHUSC). The concentration of protein, hydrolysates, and acarbose was 500 μg/mL.

The results of α‐glucosidase inhibitory activity are presented in Figure 5. LPC had an inhibition of 37.45% (500 μg/mL) which showed the high potential of lentil protein peptides in providing inhibition against α‐glucosidase. Meanwhile, hydrolysis of LPC led to a significant increase in α‐glucosidase inhibitory activity, reaching 38.80% (500 μg/mL) for LPH (*p* < .05). Indeed, sequential enzymatic hydrolysis liberated the bioactive peptides, showing higher antidiabetic activities. MTGase‐mediated cross‐linking caused the development of new peptides (LPHC) with the highest α‐glucosidase inhibitory activity among the LPC, LPH, LPHUS, and LPHUSC, reaching 40.43% inhibition (500 μg/mL). Sonication of LPH (LPHUS) led to the segregation of bioactive peptides formed by soluble aggregated peptides during hydrolysis. These new peptides did show lower α‐glucosidase inhibitory activity than LPH, LPHC, and LPHUSC. The combinatory sonication and cross‐linking of LPH led to the production of new peptides (LPHUSC) with higher α‐glucosidase inhibitory activity than LPC, LPH, and LPHUS, reaching 39.41% inhibition (500 μg/mL). While acarbose as a commercial inhibitor ((natural carbohydrate synthesized by soil bacteria) indicated stronger α‐glucosidase inhibitory activity than LPC and produced hydrolysates, reaching 50.75% inhibition (500 μg/mL; *p* < .05). It could be related to its active site‐blocking effects that efficiently inhibited the enzyme activity. The IC_50_ values were 2.58 ± 0.05, 1.74 ± 0.09, 1.20 ± 0.01, 1.75 ± 0.06, 1.23 ± 0.03, and 0.51 ± 0.07 mg/mL, respectively, for LPC, LPH, LPHC, LPHUS, LPHUSC, and acarbose (Table 2). Hence, LPHC and LPHUSC could be introduced as natural peptides to inhibit the α‐glucosidase and subsequently, control diabetes mellitus type 2.

According to recent findings, the α‐glucosidase inhibitory activity of peptides can be associated with the formation of five strong hydrogen bonds between Glu‐Ala‐Lys in the peptides and His‐674, Asp‐518, Arg‐600, Asp‐616, and Asp‐282 in α‐glucosidase, and to four hydrogen bonds between Gly‐Ser‐Arg in the peptides and residues of Asp‐282, Asp‐518, and Asp‐616 (Jiang et al., 2018; Mirzaee et al., 2022; Rezvankhah, Yarmand, & Ghanbarzadeh, 2022).

Based on the molecular docking analysis, the active site of α‐glucosidase is mainly composed of aspartic acid^518^ (ASP^518^), glutamic acid^521^ (GLU^521^), and aspartic acid^616^ (ASP^616^), and basic residues including arginine^600^ (ARG^600^) and histidine^674^ (HIS^674^; Quintero‐Soto et al., 2021). Interactions with amino acids of the α‐glucosidase catalytic site and forming unconventional hydrogen bonding between ASP^518^ and GLY^4^ (O‐H) and between ARG^600^ and LYS^3^ (O‐H) can significantly inhibit the enzyme activity. It has been reported that ARG^600^ is fundamental for α‐glucosidase inhibitory activity (Quintero‐Soto et al., 2021).

#### 3.7.2 α‐Amylase inhibitory activity

Another enzyme that is involved in glucose release in the gastrointestinal tract is α‐amylase which acts on amylose and amylopectin. Inhibition of this enzyme can control diabetes mellitus even more effectively than α‐glucosidase due to the higher MW of starch molecules than disaccharides, consequently, more glucose molecules are liberated (Karimi et al., 2020, 2021; Mirzaee et al., 2022; Rahimi et al., 2022). Acarbose has been introduced as the most efficient drug in controlling this enzyme activity while suffering from the side effects mentioned above (Casarin et al., 2021; Rezvankhah, Yarmand, & Ghanbarzadeh, 2022). Thus, the production of bioactive peptides with robust α‐amylase inhibitory activity can reduce synthetic drug consumption.

**FIGURE 6:**
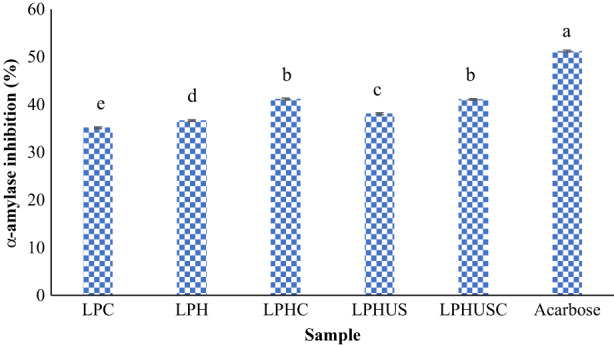
α‐amylase inhibition of LPC, hydrolyzed LPC (LPH), sonicated LPH (LPHUS), and sonicated cross‐linked LPH (LPHUSC). The concentration of protein, hydrolysates, and acarbose was 500 μg/mL.

According to Figure 6, LPC inhibited 35.13% (at 500 μg/mL) of α‐amylase activity while LPH exhibited an inhibitory activity of 36.63% (at 500 μg/mL). The inhibition of LPC could be associated with the potent bioactive peptide sequences which showed that LPC can be a good source of antidiabetic peptides (Rezvankhah, Yarmand, & Ghanbarzadeh, 2022). Enzymatic hydrolysis of LPC caused the liberation of peptides and increased the α‐amylase inhibition (Arise et al., 2019; Fadimu, Farahnaky, et al., 2022; Fadimu, Gill, et al., 2022).

MTGase‐mediated cross‐linking led to the production of new peptides (LPHC) with enhanced α‐amylase inhibition, reaching 41.16% (500 μg/mL). Cross‐linking possibly formed an aggregate of peptides which strongly indicated inhibition influences (Rezvankhah, Yarmand, & Ghanbarzadeh, 2022). The generation of peptides with new amino acid sequences is the main contributing factor for α‐amylase inhibition. Also, the MW of peptides can have a fundamental role in substituting at the active site of the enzyme and exerting inhibition impacts.

Sonication also increased the α‐amylase inhibition compared to LPC and LPH (*p* < .05). Disruption of soluble aggregated peptides led to the release of peptides with specific amino acid sequences and exhibited α‐amylase inhibition of 38.06% by LPHUS (500 μg/mL). Similar results were found for lupin protein hydrolysates pretreated with ultrasonic (Fadimu, Farahnaky, et al., 2022). In vitro biological assays indicated that α‐amylase inhibitory activity was significantly improved in sonicated hydrolysates compared with nonsonicated sample (Fadimu, Farahnaky, et al., 2022).

The combined sonication and MTGase‐mediated cross‐linking treatments led to α‐amylase inhibition of 41.06% (500 μg/mL) for LPHUSC (Figure 6). Acarbose as the commercial inhibitor showed the highest α‐amylase inhibitory activity. The IC_50_ values of LPC, LPH, LPHC, LPHUS, LPHUSC, and acarbose were 4.2 ± 0.12, 2.5 ± 0.08, 1.35 ± 0.07, 1.95 ± 0.11, 1.16 ± 0.05, and 0.43 ± 0.02 mg/mL, respectively (Table 2). These values showed that cross‐linking by MTGase and combined treatments of cross‐linking and sonication led to the generation of bioactive lentil peptides with efficient inhibition of α‐amylase. Hence, LPHC and LPHUSC could be introduced as natural peptides to control diabetes mellitus type 2.

Based on the molecular docking analysis obtained for α‐amylase and purified peptides from chickpea protein hydrolysis by Alcalase, it was reported that the activity of α‐amylase could be reduced up to one million times when replacing/changing/blocking ASP^197^, whereas the substitution of GLU^233^ and ASP^300^ could decrease the enzyme efficiency a thousand times (Quintero‐Soto et al., 2021). The differences in enzyme inhibition could be due to interactions (electrostatic, hydrogen bridge, and hydrophobic) and also the stability of the interactions (Quintero‐Soto et al., 2021). Based on the reports, the hydrophobic interactions more efficiently contribute to stabilizing the inhibitor–enzyme complex with low binding energy (Quintero‐Soto et al., 2021).

### 3.8 Umami taste development

Umami taste is derived from specific amino acid composition. Aspartic and glutamic acids are the main contributing amino acids that confer umami taste (Habinshuti et al., 2019; Rezvankhah et al., 2021a; Wang et al., 2020). LPC and the produced hydrolysates have been found as the rich source of aspartic and glutamic acids (Rezvankhah et al., 2021a, 2021b; Rezvankhah, Yarmand, & Ghanbarzadeh, 2022). Hydrolysis has been shown to release peptides with umami taste developing amino acid residues (Song et al., 2013). Peptides with MW in the range of 1–3 kDa produced by cross‐linking of hydrolysates have shown an impressive effect on the development of umami taste (Song et al., 2013; Yan et al., 2021; Zhang, Cheng, et al., 2021). Moreover, it has been reported that cross‐linking has masking effects on the bitterness of soy peptides (Zhang, Cheng, et al., 2021).

**FIGURE 7:**
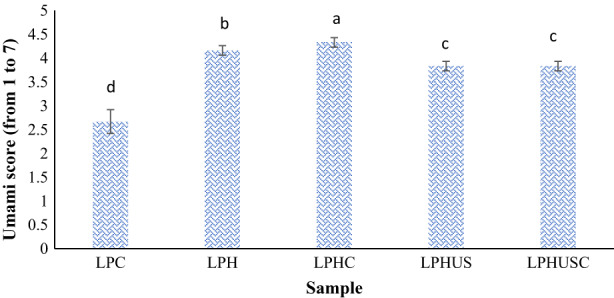
Umami taste scores of LPC, hydrolyzed LPC (LPH), sonicated LPH (LPHUS), and sonicated cross‐linked LPH (LPHUSC).

According to Figure 7, LPC had an umami taste due to its umami amino acid profile as reported in previous studies (Rezvankhah et al., 2021a, 2021b). Hydrolysis increased the umami taste score due to the liberation of peptides (LPH) with enhanced umami contributing amino acid residues (Sonklin et al., 2018; Rezvankhah et al., 2021a; Yan et al., 2021; Wang et al., 2022). On the other hand, the hydrolysates with low MW (Figure 2) have shown the strongest effect on saltiness and umami enhancement (Wang et al., 2022; Wei et al., 2018; Yan et al., 2021). It was also shown that the resulting peptides from flaxseed protein hydrolysates with an MW higher than 1000 Da could improve the mouthfulness and stability in umami soup, whereas peptides with MW of 128–1000 Da mainly contributed to the generation of meat‐like flavor compounds with a significant effect on umami taste and bitterness (Wei et al., 2018). Also, Rezvankhah et al. (2021a) reported that sequential hydrolysis of lentil protein led to the liberation of peptides with MW less than 4 kDa which have been suggested as the most contributing peptides in umami and meaty flavors.

The highest score was given to the LPHC which might have higher umami peptides and amino acids (Table 1). Umami scores of LPHUS and LPHUSC were not significantly different (*p* > .05). It has been reported that cross‐linking of soy protein hydrolysates by MTGase has led to the production of peptides with enhanced umami taste compared with sole hydrolysates (Song et al., 2013). These authors also reported that the association of peptides by cross‐linking masked the bitterness of peptides while increasing the umami and meaty analog tastes (Song et al., 2013).

## 4 CONCLUSIONS

Hydrolysis of LPC resulted in the production of bioactive peptides with stronger antioxidant, antihypertensive, and antidiabetic activities, and also umami taste. The amino acid composition and MW distribution had impressive effects on the antioxidant activity, ACE, α‐glucosidase, and α‐amylase inhibitory activities, and also umami taste. Also, sonication and cross‐linking may generate new peptides with enhanced biological and sensory properties. Eventually, LPH, LPHC, and LPHUSC could be considered as antioxidant, antihypertensive, antidiabetic, and also meaty/umami flavor enhancers.

## CONFLICT OF INTEREST STATEMENT

The authors have declared no conflict of interest.

## Data Availability

The data supporting the results of this study were provided in the form of tables and figures. All authors state that additional data will be made available upon request to the corresponding author.
